# Supplementary figures and images for: Genome-wide identification and characterization of SPXdomain-containing genes family in eggplant
Source: PeerJ. 2024 May 28;12:e17341. doi: 10.7717/peerj.17341 (PMC11141551; doi:10.7717/peerj.17341)

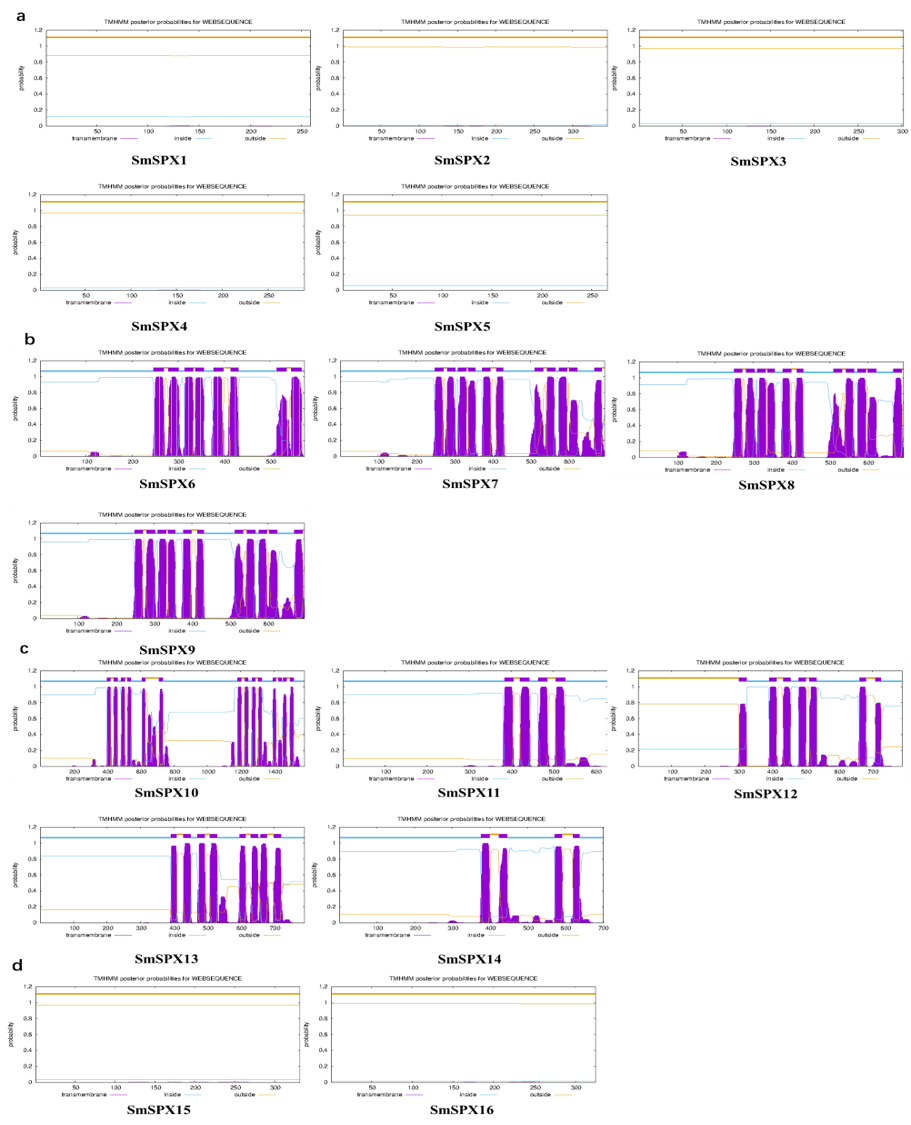

Supplement: Figure S1 — (a)SmSPX1-5 proteins transmembrane structure prediction, no transmembrane domain. (b) SmSPX6-9 proteins transmembrane structure prediction. (c) SmSPX10-14 proteins transmembrane structure prediction. (d) SmSPX15-16 proteins transmembrane structure prediction no transmembrane domain. [file peerj-12-17341-s001.png]

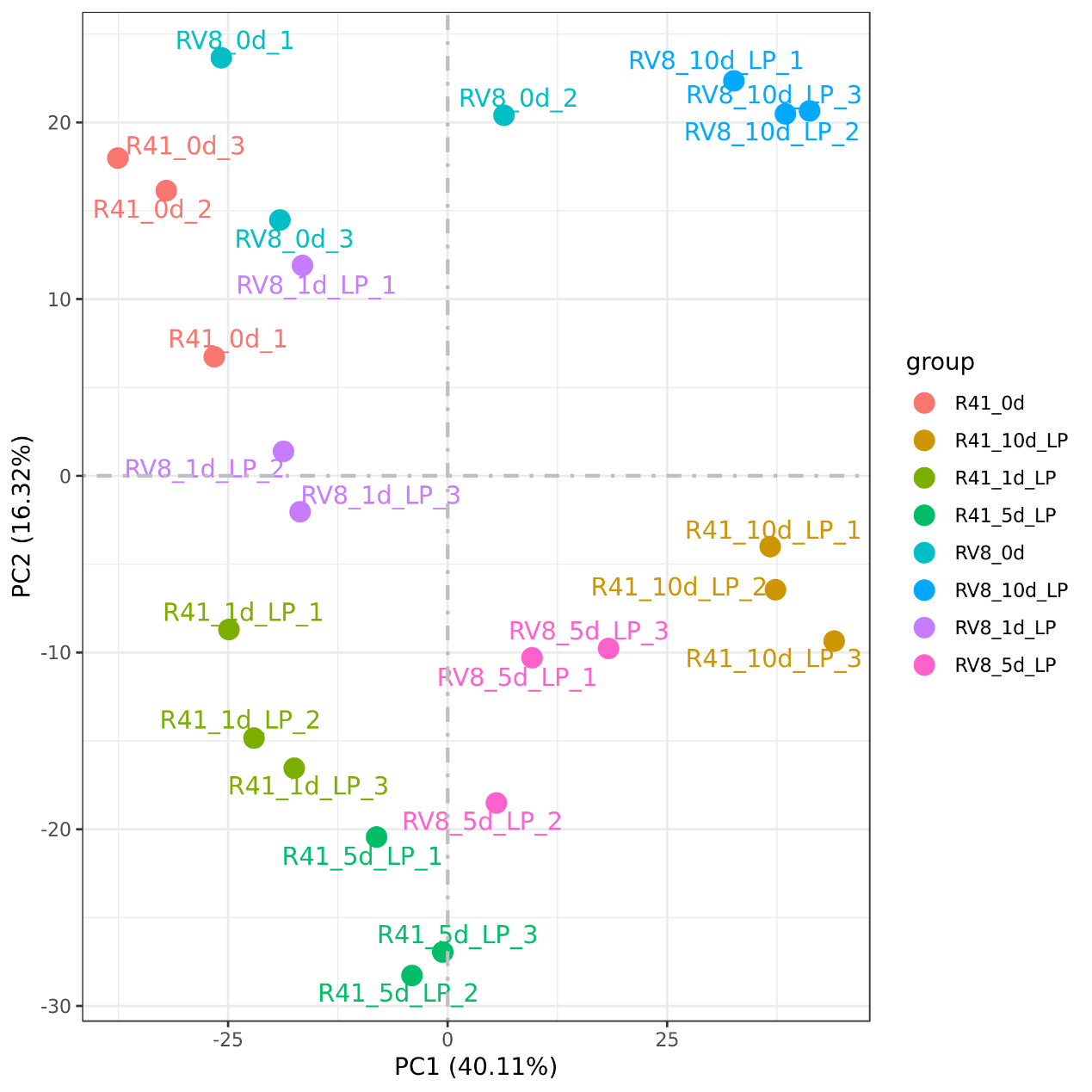

Supplement: Figure S2 [file peerj-12-17341-s002.png]

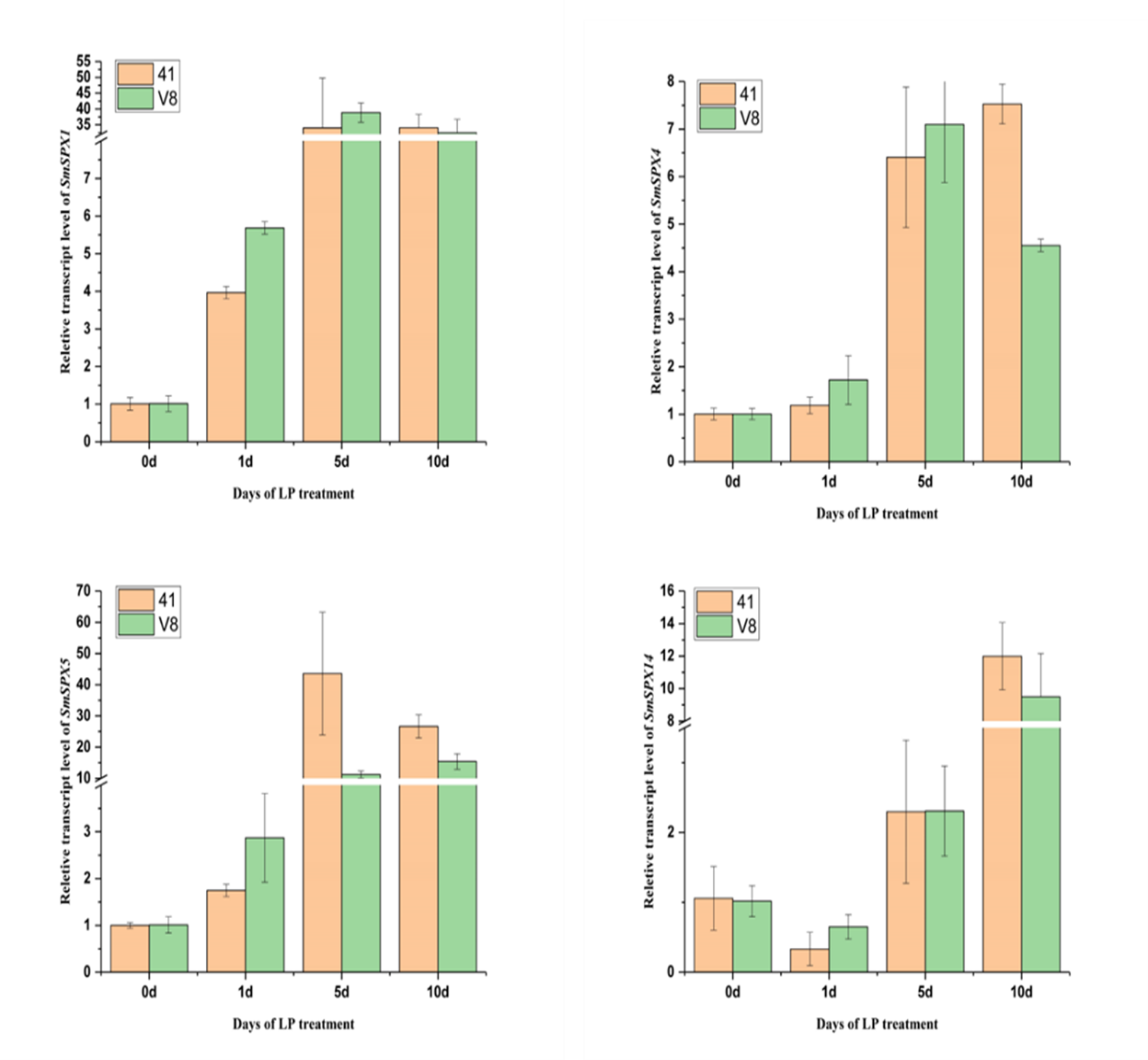

Supplement: Figure S3 — Different colors indicate different expression level, the highest in red and the lowest in green. [file peerj-12-17341-s003.png]

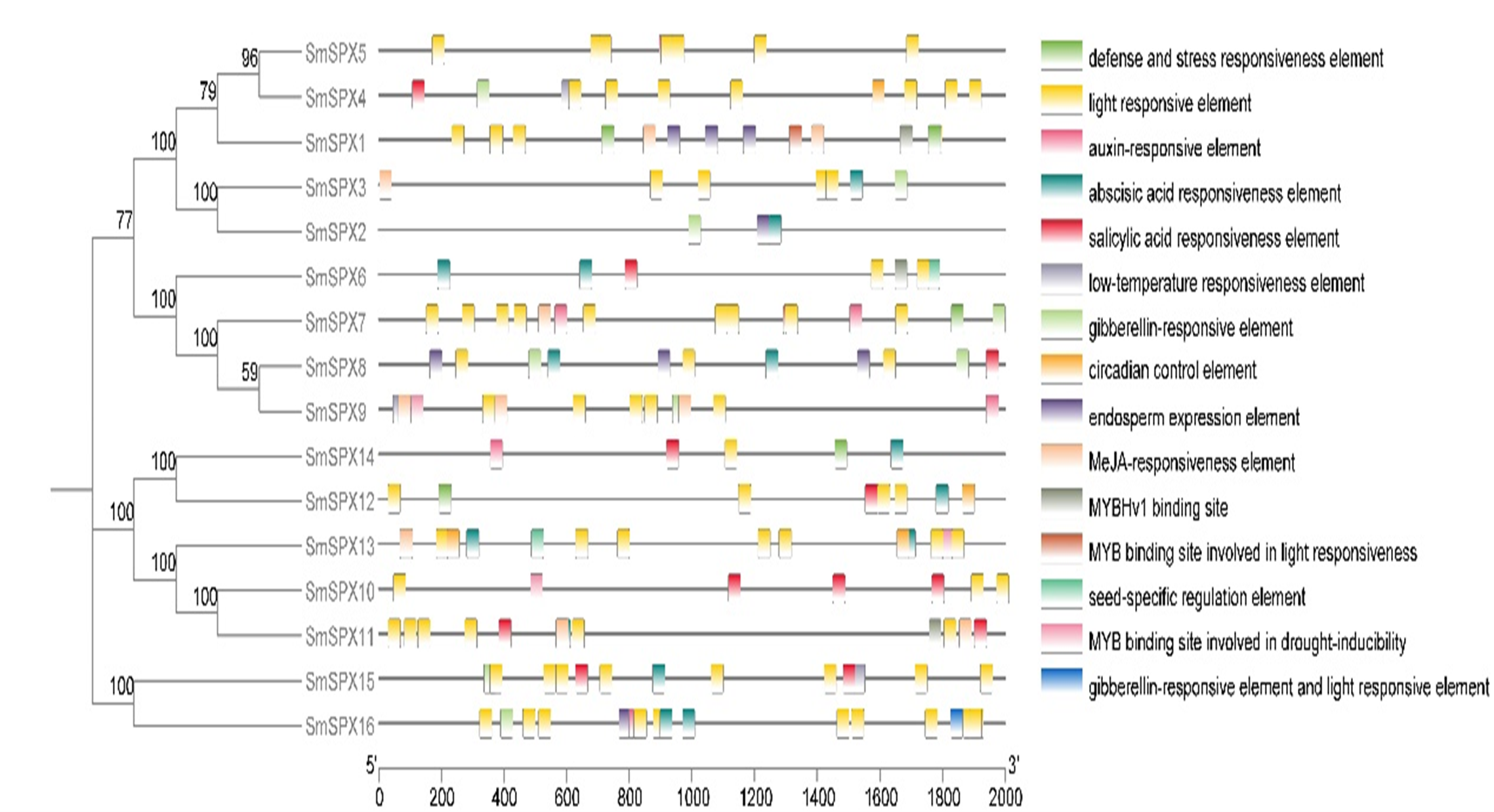

Supplement: Figure S4 — Cis-regulatory elements in the 2 kb upstream region of 16 SPX domain-containing genes coding sequences. Rounded rectangles with different colors indicate different cis-acting elements. [file peerj-12-17341-s004.png]
