# Supplementary material for: Genome-wide identification and characterization of SPXdomain-containing genes family in eggplant
Source: PeerJ. 2024 May 28;12:e17341. doi: 10.7717/peerj.17341 (PMC11141551; doi:10.7717/peerj.17341)
Supplement: Table S1 [file peerj-12-17341-s005.docx]

| qSmSPX1 5' | TTCAGCAGCCATTCTTCACA |
| --- | --- |
| qSmSPX1 3' | TATTCCTTCCCCTGCAACTC |
| qSmSPX2 5' | GATTGTTGCGTCTGCCTTTT |
| qSmSPX2 3' | CCCGGTGGAATTGTTGTATC |
| qSmSPX3 5' | CAGGGACTGAAGAAGGCAAG |
| qSmSPX3 3' | TCACTACGAGAGGCAACCAA |
| qSmSPX4 5' | TGCCGGAGATTCTGATAAGG |
| qSmSPX4 3' | TGCCACTCTATCTTGCAACT |
| qSmSPX5 5' | GGAGCTTATTCGATTGCCTTT |
| qSmSPX5 3' | GCCACTGCCTTTTGCTACTT |
| qSmSPX6 5' | ATGGCTCTTGCATGGTTTCT |
| qSmSPX6 3' | TTCCCACTATTGGCTTTTGG |
| qSmSPX7 5' | GATTAGGGGCAGTTGTTGGA |
| qSmSPX7 3' | GTGAACGTTGGACGGAAAAG |
| qSmSPX8 5' | ATTCAAGCACGTGGGGTTAG |
| qSmSPX8 3' | TGGAAAGGAAGAGCTGGATG |
| qSmSPX9 5' | CAGGCTTCAGCTGGATTTGT |
| qSmSPX9 3' | TCGGTTTCCATAGCAGGTTC |
| qSmSPX10 5' | TGCTCTTACAATGCGGACAC |
| qSmSPX10 3' | GGGGTTTTTCGAATTCCTTT |
| qSmSPX11 5' | AATATTGATCCCGCAACACC |
| qSmSPX11 3' | TCTTGGAGAATGCCAACACA |
| qSmSPX12 5' | TATTCCAGCCACAACACCAA |
| qSmSPX12 3' | GTTTTGAGCAAGCCAAGTCC |
| qSmSPX13 5' | GGATCAAGCACAGGCATACA |
| qSmSPX13 3' | ACCAACACCTCCCAAGTCAA |
| qSmSPX14 5' | TGTTTCCTAGCGCTTTTCGT |
| qSmSPX14 3' | AACCATAATCGAACGCTTGC |
| qSmSPX15 5' | TTGCTTGGATACCGTGTTTG |
| qSmSPX15 3' | GGCATTTTTCCTTGGGATTT |
| qSmSPX16 5' | CATGATGCAAGAGTGCCAAA |
| qSmSPX16 3' | AGCCCCCAGTTCTATCAACC |
|  |  |
| SmSPX1 5' | ATGAAGTTTGGGAAGAGATTGAAGC |
| SmSPX1 3' | TGGTATTGGTATAGGAGAATTGAGCT |
| SmSPX5 5' | ATGAAGTTTTGGAAAATATTGAAGAGC |
| SmSPX5 3' | CTTTGCTTCTTGTATGACCACTGG |
| SmSPX6 5' | ATGGTTGCTTTCGGGAAAAAG |
| SmSPX6 3' | CCTCTCCTCAAATATGTTGCTGAG |
| SmSPX8 5' | ATGGTGTCTTTTGGGAAAAAGTTG |
| SmSPX8 3' | ATAGAGAGAATTGTAGGTCCAAAAGGT |
| SmSPX10 5' | ATGAAATTTGGGAAAGAATTTGCT |
| SmSPX10 3' | CATACTCTTGTCTTCATCGTAGTTGAAA |
| SmSPX13 5' | ATGAAGTTCGGTAAGCAATTCAAA |
| SmSPX13 3' | TTTTTCTTTGTGCTCATCGGC |
| SmSPX15 5' | ATGAAATTTTGCAAGAAATATGAGGA |
| SmSPX15 3' | GACGCCAATAAATGCTCGACA |
| SmSPX16 5' | ATGAAGTTTGGAGAAACTTTCATGG |
| SmSPX16 3' | AAAACCAATAGCATATCTTGTTTGGT |
