# Supplementary material for: Genome-wide identification and characterization of SPXdomain-containing genes family in eggplant
Source: PeerJ. 2024 May 28;12:e17341. doi: 10.7717/peerj.17341 (PMC11141551; doi:10.7717/peerj.17341)
Supplement: Table S2 [file peerj-12-17341-s006.docx]

| Solyc00g149970 | *SlSPX1* |
| --- | --- |
| Solyc01g090890 | *SlSPX2* |
| Solyc01g091870 | *SlSPX3* |
| Solyc02g067160 | *SlSPX4* |
| Solyc02g088210 | *SlSPX5* |
| Solyc02g088230 | *SlSPX6* |
| Solyc02g088220 | *SlSPX7* |
| Solyc02g088250 | *SlSPX8* |
| Solyc05g010060 | *SlSPX9* |
| Solyc05g013180 | *SlSPX10* |
| Solyc08g007800 | *SlSPX11* |
| Solyc08g060920 | *SlSPX12* |
| Solyc08g068240 | *SlSPX13* |
| Solyc08g080200 | *SlSPX14* |
| Solyc09g075040 | *SlSPX15* |
| Solyc09g090360 | *SlSPX16* |
| Solyc11g045230 | *SlSPX17* |
| Solyc12g009480 | *SlSPX18* |
| Solyc12g056440 | *SlSPX19* |
