# Supplementary material for: Genome-wide identification and characterization of SPXdomain-containing genes family in eggplant
Source: PeerJ. 2024 May 28;12:e17341. doi: 10.7717/peerj.17341 (PMC11141551; doi:10.7717/peerj.17341)
Supplement: Table S3 — Sixteen SPX-domain-containing genes palmitoylation prediction site [file peerj-12-17341-s007.docx]

| ID |  | Position | Peptide | Score | Cutoff | Type |  |
| --- | --- | --- | --- | --- | --- | --- | --- |
| SMEL4.1_02g009290.1.01 | SmSPX2 | 4 | ****MKFGKEFSTHL | 5.802 | 5.686 | 0.394 | N-Myristoylation: Non-consensus |
| SMEL4.1_03g024350.1.01 | SmSPX4 | 188 | LYKLVKECENLIDHL | 1.505 | 1.079 | 0.152 | S-Palmitoylation: Cluster A |
| SMEL4.1_01g016630.1.01 | SmSPX6 | 135 | LKKFDKRCGYRFTNY | 2.897 | 2.293 | 0.869 | S-Palmitoylation: Cluster B |
| SMEL4.1_05g022980.1.01 | SmSPX7 | 689 | IASIFATCRTYNSLY | 4.575 | 1.323 | 0.219 | S-Geranylgeranylation: Non-consensus |
| SMEL4.1_05g022980.1.01 | SmSPX7 | 604 | YSVPQYVCSGLLMFV | 1.475 | 1.079 | 0.049 | S-Palmitoylation: Cluster A |
| SMEL4.1_05g022980.1.01 | SmSPX7 | 348 | LLLGRIFCGLGSARA | 1.377 | 1.072 | 0.215 | S-Palmitoylation: Cluster C |
| SMEL4.1_08g001320.1.01 | SmSPX8 | 602 | YSVPQYVCSGLLLFV | 1.325 | 1.079 | 0.057 | S-Palmitoylation: Cluster A |
| SMEL4.1_08g022180.1.01 | SmSPX9 | 690 | IASIIATCYTYNSLY | 4.079 | 1.323 | 0.221 | S-Geranylgeranylation: Non-consensus |
| SMEL4.1_02g028360.1.01 | SmSPX10 | 1343 | RCVWRCLCAPLYKVT | 2.507 | 2.293 | 0.7 | S-Palmitoylation: Cluster B |
| SMEL4.1_02g028360.1.01 | SmSPX10 | 194 | RSQAAGTCLITPSHD | 2.295 | 2.293 | 0.595 | S-Palmitoylation: Cluster B |
| SMEL4.1_02g028370.1.01 | SmSPX11 | 600 | RFKQLGVCNSMSATM | 2.481 | 2.293 | 0.847 | S-Palmitoylation: Cluster B |
| SMEL4.1_02g028370.1.01 | SmSPX11 | 265 | TLKNVIKCPKANSKF | 1.372 | 1.072 | 0.006 | S-Palmitoylation: Cluster C |
| SMEL4.1_09g022670.1.01 | SmSPX12 | 402 | FVGLFTGCFVTLFSV | 1.744 | 1.079 | 0.012 | S-Palmitoylation: Cluster A |
| SMEL4.1_10g015110.1.01 | SmSPX13 | 201 | FQILHCQCADTGDMP | 3.515 | 2.293 | 0.296 | S-Palmitoylation: Cluster B |
| SMEL4.1_10g017040.1.01 | SmSPX14 | 10 | KFSKQFEGQLIPEWK | 6.117 | 5.686 | 0.804 | N-Myristoylation: Non-consensus |
| SMEL4.1_09g018170.1.01 | SmSPX15 | 326 | KEHWESQCRAFIGV* | 6.577 | 3.71 | 0.457 | S-Farnesylation: Non-consensus |
| SMEL4.1_09g018170.1.01 | SmSPX15 | 4 | ****MKFCKKYEEFM | 3.45 | 1.072 | 0.029 | S-Palmitoylation: Cluster C |
| SMEL4.1_09g018170.1.01 | SmSPX15 | 326 | KEHWESQCRAFIGV* | 2.54 | 1.079 | 0.016 | S-Palmitoylation: Cluster A |
| SMEL4.1_09g018170.1.01 | SmSPX15 | 35 | LKKILKKCRKCRQSP | 2.08 | 1.072 | 0.006 | S-Palmitoylation: Cluster C |
| SMEL4.1_09g018170.1.01 | SmSPX15 | 38 | ILKKCRKCRQSPTVG | 1.532 | 1.072 | 0.052 | S-Palmitoylation: Cluster C |
| SMEL4.1_09g018170.1.01 | SmSPX15 | 228 | KLDIDLTCSICLDTV | 1.489 | 1.079 | 0.235 | S-Palmitoylation: Cluster A |
| SMEL4.1_09g018170.1.01 | SmSPX15 | 251 | GHIFCYICACKGASV | 1.156 | 1.072 | 0.334 | S-Palmitoylation: Cluster C |
| SMEL4.1_12g008960.1.01 | SmSPX16 | 4 | ****MKFGETFMEYL | 5.989 | 5.686 | 0.321 | N-Myristoylation: Non-consensus |
| SMEL4.1_12g008960.1.01 | SmSPX16 | 236 | FNPYALGCGHLFCKS | 1.272 | 1.072 | 0.498 | S-Palmitoylation: Cluster C |
