# Supplementary material for: Genome-wide identification and characterization of SPXdomain-containing genes family in eggplant
Source: PeerJ. 2024 May 28;12:e17341. doi: 10.7717/peerj.17341 (PMC11141551; doi:10.7717/peerj.17341)
Supplement: Supplemental Information 11 — To prove the validity of fluorescence quantitative test data and the quality of test materials [file peerj-12-17341-s011.docx]

1. **Experimental design**

**Definition of experimental and control group**s

Experimental:1d,5d,10d LP group and 1d,5d,10d LP+IAA group

control group:0d ,no treatment

reference genes β-actin gene（GenBank:JX524155.1）

**Number within each group**

Each group consisted of five independent plants, each of which took the same quality tissue from the same part and mixed it as a treated sample.

1. **Sample**

**Description**

All samples are from the eggplant varieties described on page 8, line 201, including roots, stems, leaves, flowers, fruits, and seeds.

**Microdissection or macrodissection、**

Microdissection

**Processing procedure**

The required sample is cut using a blade and then quickly ground to a powder in liquid nitrogen.

**If frozen, how and how quickly?**

Freeze liquid nitrogen for immediate treatment

**If fixed, with what and how quickly?**

No fixed sample

**Sample storage conitions and duration**

-80 ° C Storage time should not exceed half a month

**3. Nucleic acid extraction**

**Procedure and/or instrumentation，name of kit and details of any modifications,** **RNA integrity: method/instrument**

For details, see page 9, lines 217-231

**Details of DNase or RNase treatment**

Use consumables in direct contact with RNA, such as nuclease-free pipette heads and centrifuge tubes

**Contamination assessment (DNA or RNA)Nucleic acid quantification, Instrument and method**

RNA concentration and mass were measured using an ultramicrospectrophotometer, page 9, line 222

**RIN/RQIor C of 3' and 5' transcripts,** **concentration**

| **Sample** | **RIN** | **Concentration(ng/ul)** |
| --- | --- | --- |
| **0d** | **7.6** | **408.2** |
| **1d LP** | **8.8** | **508.7** |
| **1d LP+IAA** | **6.8** | **349.2** |
| **5d LP** | **9.1** | **607.6** |
| **5d LP+IAA** | **7.6** | **529.7** |
| **10d LP** | **7.9** | **799.7** |
| **10d LP+IAA** | **8.2** | **575.9** |

**4.Reverse transcription**

**Complete reaction conditions**

Using Mona Bio's one-step reverse transcription kit, RNA, nuclease-free water genome removal enzyme and RTlll Mix reagent were added for 5 minutes at 37 ℃, 15 minutes at 55 ℃, and 5 minutes at 85 ℃.

**Amount of RNA and reaction volume**

| **Sample** | **reaction volume(ul)** |
| --- | --- |
| **0d** | **2.44** |
| **1d LP** | **1.96** |
| **1d LP+IAA** | **2.86** |
| **5d LP** | **1.64** |
| **5d LP+IAA** | **1.88** |
| **10d LP** | **1.25** |
| **10d LP+IAA** | **1.73** |

**Priming oligonucleotide (if using GSP) and concentrationReverse transcriptase and concentration**

Primer concentration:10 pmol/ul

Reverse transcriptase concentration: ambiguity, We can provide product number: Monad,REF:MR05101

**Temperature and time**

5 minutes at 37 ℃, 15 minutes at 55 ℃, and 5 minutes at 85 ℃.

**5.qPCR target information**

**Gene symbol and Sequence accession number**

Genetic details can be found using the gene number in the SGN (https://solgenomics.net/) database, page 7, line 168

SMEL4.1_01g017740.1.01 SmSPX1

SMEL4.1_02g009290.1.01 SmSPX2

SMEL4.1_02g028380.1.01 SmSPX3

SMEL4.1_03g024350.1.01 SmSPX4

SMEL4.1_06g020500.1.01 SmSPX5

SMEL4.1_01g016630.1.01 SmSPX6

SMEL4.1_05g022980.1.01 SmSPX7

SMEL4.1_08g001320.1.01 SmSPX8

SMEL4.1_08g022180.1.01 SmSPX9

SMEL4.1_02g028360.1.01 SmSPX10

SMEL4.1_02g028370.1.01 SmSPX11

SMEL4.1_09g022670.1.01 SmSPX12

SMEL4.1_10g015110.1.01 SmSPX13

SMEL4.1_10g017040.1.01 SmSPX14

SMEL4.1_09g018170.1.01 SmSPX15

SMEL4.1_12g008960.1.01 SmSPX16

**Amplicon length**

SMEL4.1_01g017740.1.01 SmSPX1 136 bp

SMEL4.1_02g009290.1.01 SmSPX2 217 bp

SMEL4.1_02g028380.1.01 SmSPX3 137 bp

SMEL4.1_03g024350.1.01 SmSPX4 163 bp

SMEL4.1_06g020500.1.01 SmSPX5148 bp

SMEL4.1_01g016630.1.01 SmSPX6 107 bp

SMEL4.1_05g022980.1.01 SmSPX7 189 bp

SMEL4.1_08g001320.1.01 SmSPX8 117 bp

SMEL4.1_08g022180.1.01 SmSPX9 203 bp

SMEL4.1_02g028360.1.01 SmSPX10 151 bp

SMEL4.1_02g028370.1.01 SmSPX11 190 bp

SMEL4.1_09g022670.1.01 SmSPX12 186 bp

SMEL4.1_10g015110.1.01 SmSPX13 108 bp

SMEL4.1_10g017040.1.01 SmSPX14 127bp

SMEL4.1_09g018170.1.01 SmSPX15 131 bp

SMEL4.1_12g008960.1.01 SmSPX16 181 bp

SMEL4.1_10g011330.1.01 Smactin 200 bp

**In silico specificity screen**

Sequence comparison showed the specificity of amplification primers

**Location of each primer by exon or intron**

The locations of primer sequences can be compared in the SGN (https://solgenomics.net/) database according to the provided primer sequences.

**6.qPCR oligonucleotides**

**Primer sequences**

qSmSPX1 5' TTCAGCAGCCATTCTTCACA

qSmSPX1 3' TATTCCTTCCCCTGCAACTC

qSmSPX2 5' GATTGTTGCGTCTGCCTTTT

qSmSPX2 3' CCCGGTGGAATTGTTGTATC

qSmSPX3 5' CAGGGACTGAAGAAGGCAAG

qSmSPX3 3' TCACTACGAGAGGCAACCAA

qSmSPX4 5' TGCCGGAGATTCTGATAAGG

qSmSPX4 3' TGCCACTCTATCTTGCAACT

qSmSPX5 5' GGAGCTTATTCGATTGCCTTT

qSmSPX5 3' GCCACTGCCTTTTGCTACTT

qSmSPX6 5' ATGGCTCTTGCATGGTTTCT

qSmSPX6 3' TTCCCACTATTGGCTTTTGG

qSmSPX7 5' GATTAGGGGCAGTTGTTGGA

qSmSPX7 3' GTGAACGTTGGACGGAAAAG

qSmSPX8 5' ATTCAAGCACGTGGGGTTAG

qSmSPX8 3' TGGAAAGGAAGAGCTGGATG

qSmSPX9 5' CAGGCTTCAGCTGGATTTGT

qSmSPX9 3' TCGGTTTCCATAGCAGGTTC

qSmSPX10 5' TGCTCTTACAATGCGGACAC

qSmSPX10 3' GGGGTTTTTCGAATTCCTTT

qSmSPX11 5' AATATTGATCCCGCAACACC

qSmSPX11 3' TCTTGGAGAATGCCAACACA

qSmSPX12 5' TATTCCAGCCACAACACCAA

qSmSPX12 3' GTTTTGAGCAAGCCAAGTCC

qSmSPX13 5' GGATCAAGCACAGGCATACA

qSmSPX13 3' ACCAACACCTCCCAAGTCAA

qSmSPX14 5' TGTTTCCTAGCGCTTTTCGT

qSmSPX14 3' AACCATAATCGAACGCTTGC

qSmSPX15 5' TTGCTTGGATACCGTGTTTG

qSmSPX15 3' GGCATTTTTCCTTGGGATTT

qSmSPX16 5' CATGATGCAAGAGTGCCAAA

qSmSPX16 3' AGCCCCCAGTTCTATCAACC

qSm actin 5' GTCGGAATGGGACAGAAGGATG

qSm actin 3' GTGCCTCAGTCAGGAGAACAGGGT

**RTPrimerDB identification number**

Not Applicable
